# Gd^3+^-Doped NaYF_4_:Yb,Er Upconversion Nanoparticles: Synthesis, Luminescence Enhancement, and Anti-Counterfeiting Applications

**DOI:** 10.3390/nano16201320

**Published:** 2026-10-19

**Authors:** Yidan Liu, Jiazhan Chang, Shibiao Xie, Mingsu Si, Xianwei Zuo, Bin Liu, Xiaoping Gao, Xin Li, Zijie Wu

**Affiliations:** 1 School of Materials and Energy, Electron Microscopy Centre of Lanzhou University, Lanzhou University, Lanzhou 730000, China; 2 Key Laboratory of Sensor and Sensing Technology of Gansu Province, Institute of Sensing Technology, Gansu Academy of Sciences, Lanzhou 730000, China

**Keywords:** enhanced light emission, upconversion nanoparticles, energy migration, anti-counterfeiting technology

## Abstract

Precise control over the optical properties of lanthanide-doped upconversion nanomaterials is of fundamental importance for their wide-ranging applications. In this study, Gd^3+^-doped nanoparticles with enhanced upconversion luminescence were prepared. The influence of Gd^3+^ doping on luminescence was systematically investigated. This method significantly improves the uniformity of particle size and enhances the luminescence intensity of NaYF_4_:Yb/Er nanoparticles. Doping with Gd^3+^ replaces Y^3+^ ions and induces local lattice distortion, which modifies the crystal field symmetry around the rare-earth ions, increases their transition probability, and thereby directly enhances the luminescence intensity. Additionally, Excitation-power-dependent log–log analysis reveals that Gd^3+^ incorporation modifies the effective photon numbers of the upconversion channels. Importantly, this material enables the generation of high-resolution anti-counterfeiting patterns through inkjet printing. The proposed method to enhance upconversion emission is meaningful for designing novel luminescent materials and expanding their optical applications.

## 1. Introduction

Lanthanide-doped upconversion nanoparticles (UCNPs) have gained tremendous attention due to their remarkable properties, such as low autofluorescence background, sharp emission bands, large anti-Stokes shifts, long luminescence decay time, and high resistance to photobleaching [1,2,3,4,5,6]. They have excellent applications in biological imaging, photodynamic therapy, biosensing and detection, optoelectronic devices, and anti-counterfeiting [7,8,9,10,11,12]. However, enhancing upconversion emission intensity remains a major challenge in these fields, especially for high-resolution anti-counterfeiting applications [13,14,15,16,17,18,19,20,21,22,23,24]. NaYF_4_ nanoparticles are known for their efficient upconversion luminescence due to the minimized multiphonon-assisted relaxation processes in their emitting states. To further enhance light absorption, Yb^3+^, which has a large absorption cross-section, is commonly doped [25]. The excited state ^5^F_5/2_ of Yb^3+^ is energetically close to the ^4^I_11/2_ state of Er^3+^, enabling highly efficient energy transfer. In this system, Yb^3+^ possesses a simple two-level electronic configuration, which enables strong absorption of near-infrared (NIR) excitation light around 980 nm. The absorbed energy is subsequently transferred to adjacent Er^3+^ ions via a non-resonant process. NaYF_4_:Yb/Er is an excellent luminescent system. The UC mechanism is mainly a two-photon energy transfer upconversion (ETU) process, with potential three-photon involvement at higher powers [26,27,28]. Moreover, many reports have described various approaches to enhancing the intensity of upconversion luminescence. Sagaidachnaya et al. investigated the variation in the luminescence intensity of hydrothermally synthesized upconversion β-NaYF_4_:Yb/Er particles under different synthesis conditions. Their findings demonstrate that employing ammonium fluoride and a medium with a pH of 3 is essential for achieving the maximum luminescence intensity in these particles [29]. Alexandrov et al. demonstrated that reducing the defect density in powder particles is an effective strategy for improving the intensity of their upconversion luminescence [30]. In addition, conventional enhancement strategies for upconversion, such as lattice engineering, core–shell design, and plasmonic coupling, often suffer from compatibility issues and procedural complexity. Lanthanide doping offers an intrinsic alternative that relies on optimal sensitizer/activator selection and inert-ion modulation of the crystal field. This strategy enhances luminescence efficiently without compromising nanoparticle integrity and features superior controllability, generality, and structural noninvasiveness. Doping ions during synthesis plays a crucial role in influencing the crystallographic phase, particle size, and optical emission of UCNPs [31]. More importantly, dopants can induce strain in the crystal lattice, modulate the crystal field around the activator ions, and enhance luminescence [32,33,34,35,36,37]. Vidyakina et al. [38] systematically investigated the Gd^3+^-doping effect on upconversion emission of NaYF_4_:Yb^3+^,Er^3+^/Tm^3+^ microparticles, establishing the relationship between Gd^3+^ concentration and emission intensity. Klier & Kumke [39] demonstrated that Gd^3+^ co-doping in NaYF_4_:Yb:Er nanoparticles influences upconversion luminescence properties, with effects attributable to changes in the local crystal field and energy transfer pathways. Since the ^6^P_7/2_ level, which represents the next electronic state above the ground state of Gd^3+^, is spectroscopically found in the ultraviolet region and is therefore much higher in energy than the excited state levels of Er^3+^ and Yb^3+^, any luminescence quenching by energy transfer between these ions can be ruled out [40]. Therefore, Gd^3+^ doping is beneficial for improving luminescence performance. However, the power-dependent luminescence characteristics of Gd^3+^ doping and its application in anti-counterfeiting have rarely been investigated. We aim to incorporate Gd^3+^ into the mature NaYF_4_:Yb/Er system to further enhance its luminescence performance, and to investigate the underlying mechanism as well as its application in anti-counterfeiting.

Here, a new Yb^3+^-Gd^3+^-Er^3+^ triply doped luminescent material NaYF_4_:Yb/Er/Gd was designed, and the influence of Gd^3+^ dopant ions on luminescence was systematically studied. Compared with NaYF_4_:Yb/Er, the particle size of NaYF_4_:Yb/Er/Gd was uniform, and the green–red fluorescence ratio was significantly enhanced. In addition, the luminescence mechanism was studied, providing significant theoretical guidance for improving the performance of these materials. Finally, these highly luminescent upconversion materials have excellent luminescent effects for anti-counterfeiting. When combined with inkjet printing technology, these materials can produce complex, high-resolution upconversion luminescence patterns with fine details. This research provides new ideas for enhancing the upconversion luminescence performance and applying it to anti-counterfeiting.

## 2. Materials and Methods

### 2.1. Materials

YCl_3_·6H_2_O, YbCl_3_·6H_2_O, ErCl_3_·6H_2_O, GdCl_3_·6H_2_O, and NH_4_F were purchased from Sigma-Aldrich (Darmstadt, Germany). 1-octadecene (ODE, 90%), NaOH, and oleic acid (OA, 90%) were supplied by Thermo Fisher Scientific (Waltham, MA, USA) Alfa Aesar. Methanol, glycerol (99%), ethanol, and cyclohexane were obtained from Sinopharm (Beijing, China). All reagents were of analytical grade and used without further purification. Deionized water (18.2 MΩ·cm, 18202V AXL, Zhiang Instruments Co., Ltd., Shanghai, China) was used throughout the experiments. Data processing was performed using the original software.

### 2.2. Synthesis of Upconversion Nanoparticles

The upconversion nanoparticles NaYF_4_:Yb/Er/Gd were synthesized through thermal decomposition in the presence of oleic acid (OA) and octadecene. YCl_3_·6H_2_O (1.44 mmol), YbCl_3_·6H_2_O (0.4 mmol), ErCl_3_·6H_2_O (0.04 mmol), and GdCl_3_·6H_2_O (0.12 mmol) were dissolved in 2 mL of deionized water and added to a 100 mL flask containing 14 mL of OA and 30 mL of 1-octadecene. The mixture was stirred at room temperature for 20 min, and then gradually heated to 110 °C and maintained for 1 h. It was further heated to 160 °C for another hour under a nitrogen atmosphere to remove water. The reaction mixture was then cooled to 50 °C. Meanwhile, a methanol solution (20 mL) containing NH_4_F (8 mmol) and NaOH (5 mmol) was added to the flask and stirred at 50 °C for 1 h. The temperature was then increased to 70 °C to evaporate the methanol. After methanol removal, the mixture was heated to 110 °C and held for 1 h under nitrogen to eliminate residual oxygen and water. Subsequently, the mixture was heated to 300 °C and maintained at this temperature for 1 h. The resulting product was washed several times with ethanol and cyclohexane and finally dispersed in cyclohexane. The chemical reaction as follows: 0.72 YCl_3_ + 0.20 YbCl_3_+ 0.02 ErCl_3_+ 0.06 GdCl_3_+ 4 NH_4_F + 4 NaOH → NaY_0.72_Yb_0.2_Er_0.02_Gd_0.06_F_4_ + 3 NaCl + 4 NH_4_OH (for the 6 mol% Gd sample).

### 2.3. Instrumentation

High-angle annular dark-field STEM (HAADF-STEM) images were obtained using an aberration-corrected transmission electron microscope (TEM; Titan Themis Cubed G2 300, Thermo Fisher Scientific, Waltham, MA, USA) operating at 300 kV to examine the microscopic morphology. XRD was performed with a Bruker D8 Advance diffractometer (Bruker, Billerica, MA, USA) using Cu-Kα radiation (λ = 1.5406 Å), scanning from 10° to 80° at a rate of 2°/min, under a constant voltage of 40 kV. The elemental composition of the as-prepared UCNP was determined using ICP-OES (iCAP PRO, Thermo Fisher Scientific, Waltham, MA, USA). Upconversion emission spectra were recorded using an FS5 fluorescence spectrometer (Edinburgh Instruments Ltd., Livingston, UK). The measurements were performed under the following conditions: front-face excitation geometry with 90° collection, laser beam diameter of ~3 mm, spectral resolution of 1 nm, scanning speed of 2000 nm/min, acquisition time of 0.2 s per step, and wavelength range of 200–700 nm. The spectra were corrected for the wavelength-dependent spectral response of the detector using a calibrated standard lamp. The excitation power density was estimated to be ~7.1 W/cm^2^ at a total laser power of 0.5 W. Printing was performed using an EHDJet-H inkjet printer (Wuhan Huaweike Intelligent Technology Co., Ltd., Wuhan, China).

### 2.4. Preparation of Printing Ink

The synthesized NaYF_4_:Yb/Er/Gd nanoparticles were dispersed in a cyclohexane–glycerol mixture with a volume ratio of 9:1 to prepare the printing ink. The mixture was stirred for 10 min and then sonicated for 15 min to ensure complete dispersion of the nanoparticles.

## 3. Results

### 3.1. The Influence of Shape, Structure, and Luminescence Intensity Before and After Doping with Gd^3+^ in NaYF4:Yb/Er

The Gd^3+^ doping amount was varied, while the total ion content (Y^3+^, Yb^3+^, Er^3+^, Gd^3+^) and the Yb^3+^ and Er^3+^ concentrations were held constant. According to previous research [41,42], controlling the doping concentrations of Yb^3+^ and Er^3+^ at 20% and 2% respectively was the most suitable concentration ratio for luminescence. To further determine the rare-earth element content in the obtained nanoparticles, the elemental composition of the as-prepared UCNPs was analyzed and the results are listed in Table 1. For this, 5 mg of the as-prepared UCNP was dissolved in 5 mL of HNO_3_. The elemental composition of the as-prepared UCNP was determined using inductively coupled plasma atomic emission spectroscopy (ICP-OES). It can be noted that the percentage of rare-earth ions present in the sample does not deviate much from the initial amount involved in the reaction.

**Figure 1:**
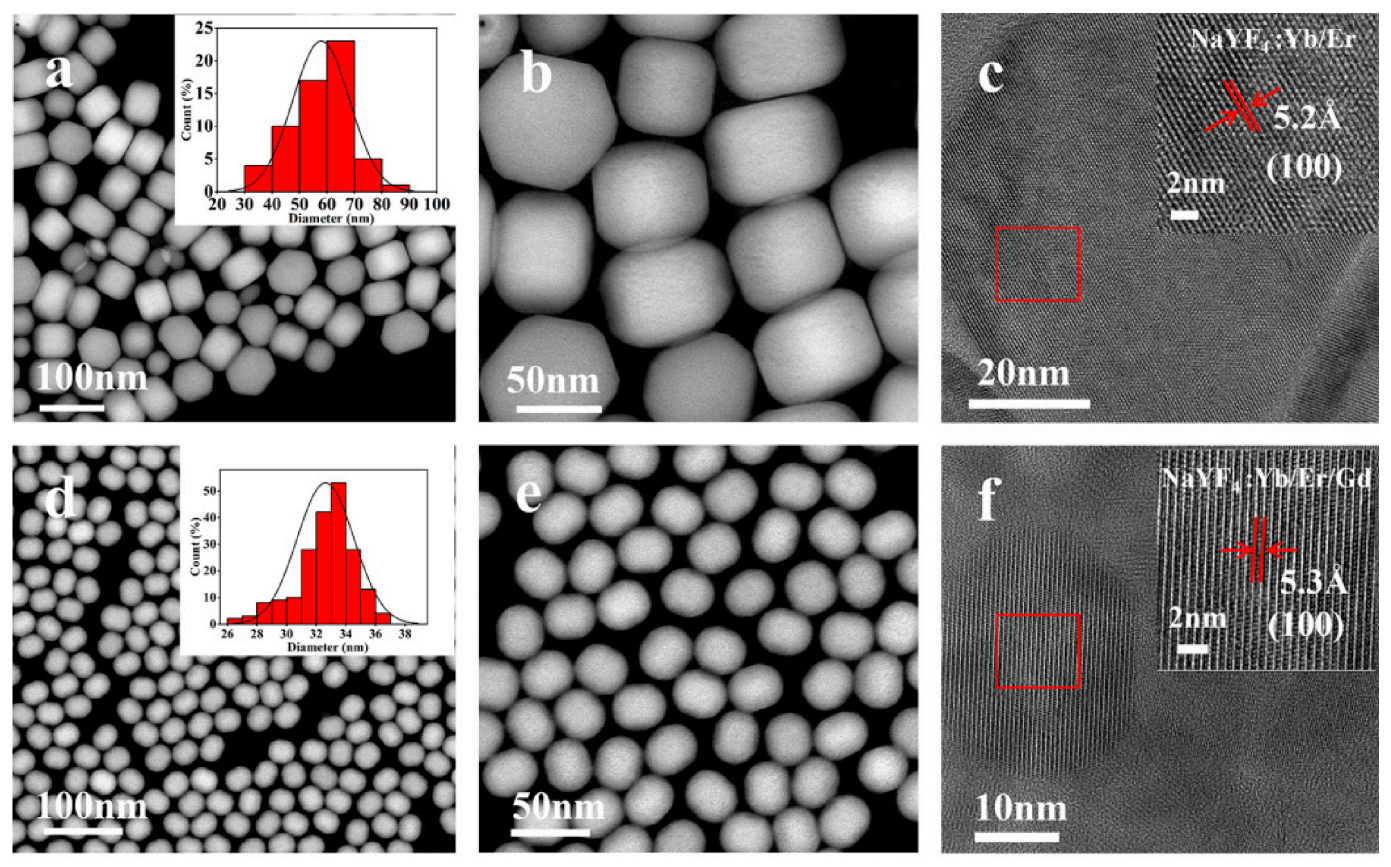
(**a**) STEM image and corresponding particle-size histogram of NaYF_4_:Yb/Er nanoparticles. (**b**) A zoomed-in view of (**a**). (**c**) HRTEM image of NaYF_4_:Yb/Er nanocrystals. (**d**) STEM image and corresponding particle-size histogram of NaYF_4_:Yb/Er/Gd nanoparticles. (**e**) A zoomed-in view of (**d**). (**f**) HRTEM image of NaYF_4_:Yb/Er/Gd nanocrystals.

Next, changes in the morphology, structure, and luminescence intensity of NaYF_4_:Yb/Er upconversion materials before and after Gd^3+^ doping were systematically characterized and studied. The morphology of NaYF_4_:Yb/Er (20/2 mol%) nanoparticles was characterized using STEM, as shown in Figure 1a,b. The TEM images and corresponding particle-size histogram reveal that the particles are largely agglomerated and exhibit a broad size distribution. The average particle size is approximately 60 nm, as shown in the inset of Figure 1a. The high-resolution TEM (HRTEM) image in Figure 1c shows distinct (100) crystal planes with an interplanar spacing of 0.52 nm.

When Gd^3+^ was doped into the NaYF_4_:Yb/Er system, the resulting nanoparticles exhibited significantly improved dispersion, with a uniform size distribution and no visible agglomeration (Figure 1d,e). The average particle size was determined by analyzing approximately 200 particles from the TEM images. This analysis yielded an average size of around 30 nm, presented in the inset of Figure 1d. The HRTEM image in Figure 1f indicates a slight increase in the spacing of the (100) planes. The increased d-spacing (from ~5.2 Å to ~5.3 Å) indicates lattice expansion from Gd^3+^ substitution, consistent with the larger ionic radius of Gd^3+^ compared to Y^3+^. The substitution of Y^3+^ ions by heavier Gd^3+^ ions increases the surface electron charge density, thereby reducing the diffusion of negatively charged F^−^ ions to the surface [35]. This enhanced coulomb repulsion between F^−^ ions and the negatively charged surface significantly limits surface diffusion.

**Figure 2:**
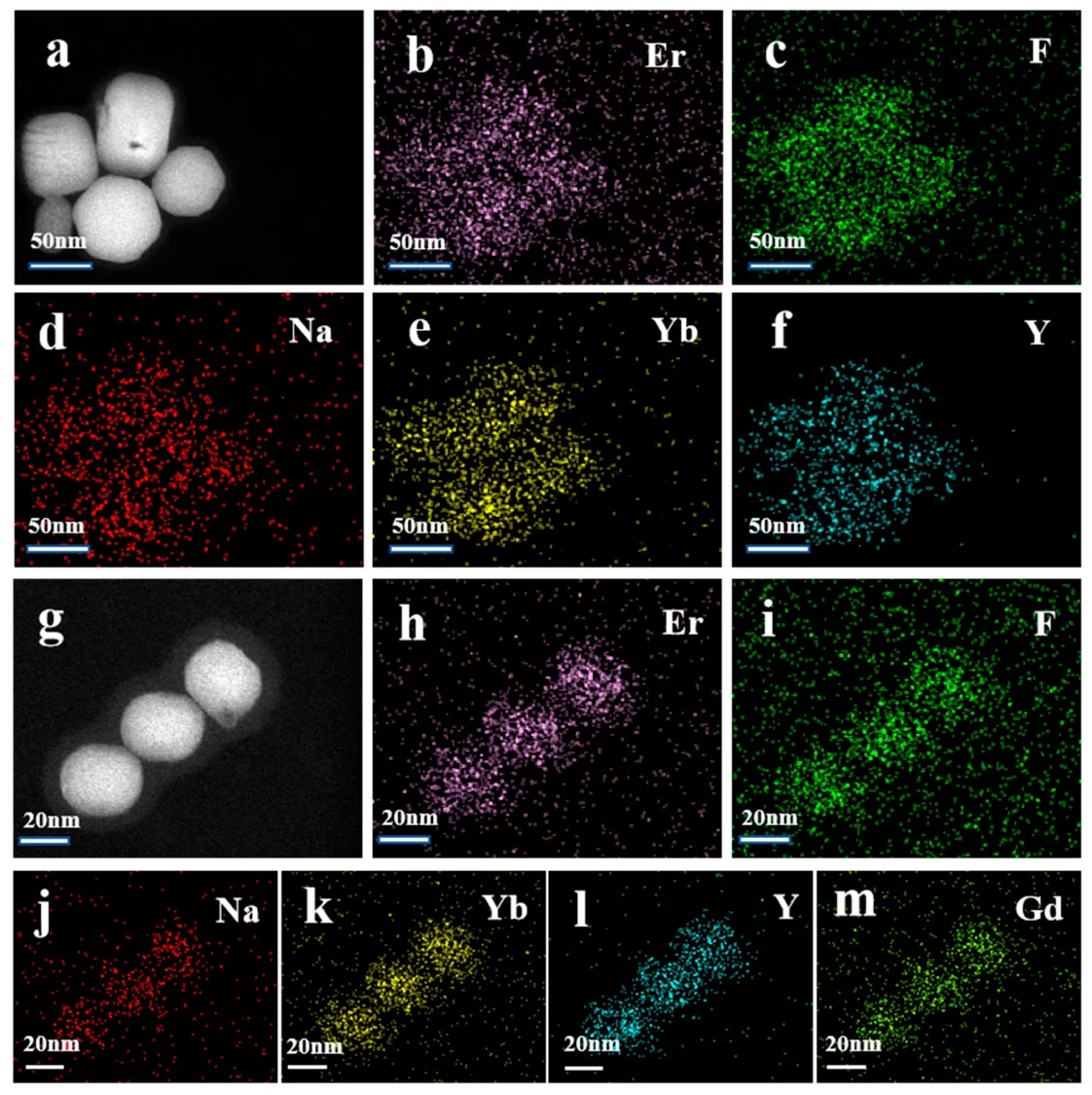
(**a**) HAADF-STEM image and (**b**–**f**) EDX elemental mapping images of NaYF_4_:Yb/Er nanoparticles. (**g**) HAADF-STEM image and (**h**–**m**) corresponding EDX elemental mapping images of NaYF_4_:Yb/Er nanoparticles doped with Gd^3+^.

Chemical composition analysis was performed using HAADF-STEM imaging and energy-dispersive X-ray spectroscopy (EDX) mapping. The elemental distributions of Er, F, Na, Yb, and Y for the nanoparticles shown in Figure 2a are presented in Figure 2b–f, respectively. It can be observed that the Er, F, Na, Yb, and Y elements are uniformly distributed across the nanoparticles. EDX mapping of Gd^3+^-doped NaYF_4_:Yb/Er nanoparticles was conducted to further examine the incorporation of Gd^3+^. Figure 2g shows the corresponding HAADF-STEM image, while Figure 2h–m show EDX elemental maps of Er, F, Na, Yb, Y, and Gd, respectively. The results confirm that Gd is uniformly distributed throughout the nanoparticles.

**Figure 3:**
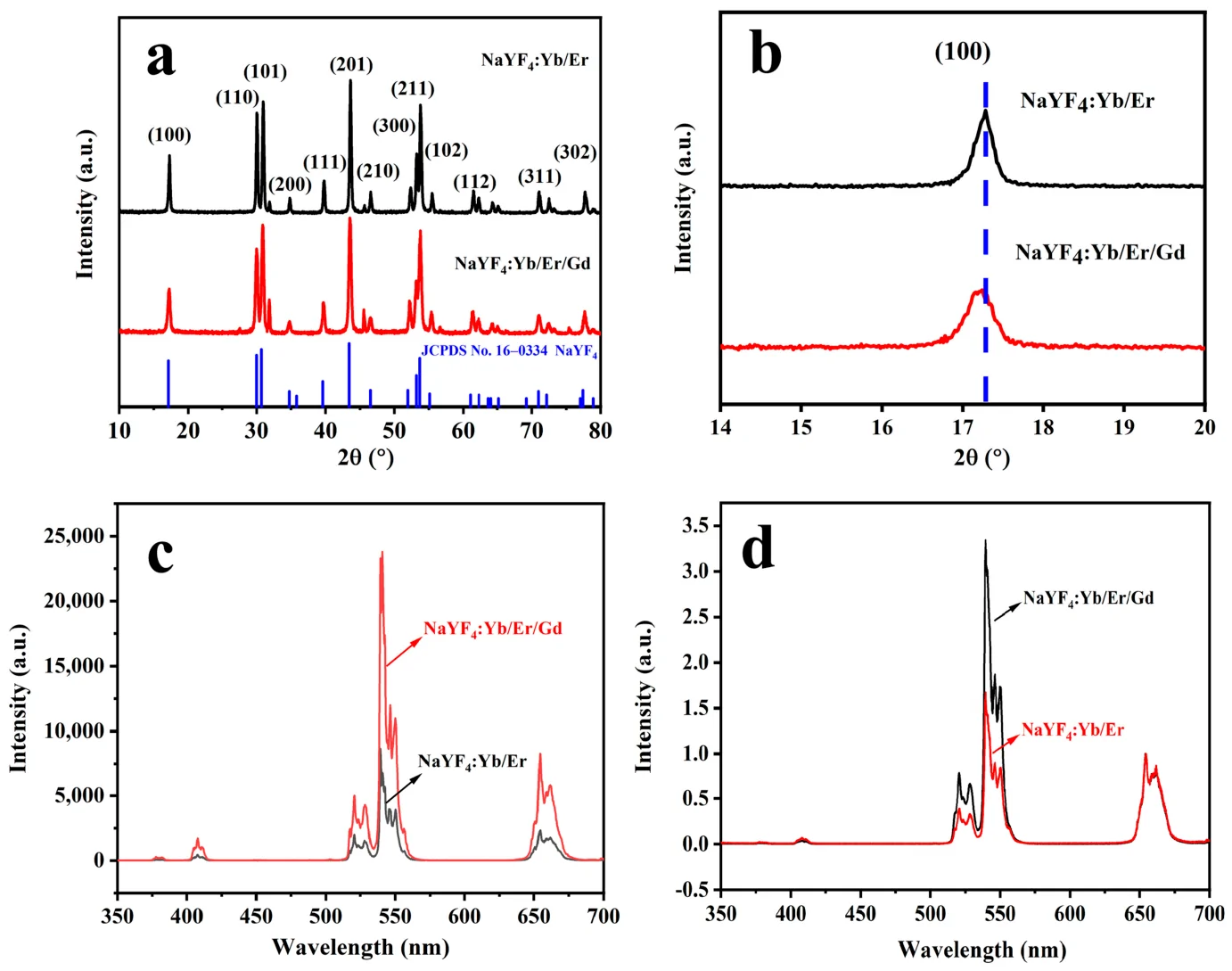
(**a**) XRD patterns of NaYF_4_:Yb/Er/Gd. (**b**) Enlarged XRD pattern of NaYF_4_:Yb/Er/Gd. (**c**) Upconversion emission spectra of NaYF_4_:Yb/Er and NaYF_4_:Yb/Er doped with Gd^3+^. (**d**) Upconversion emission spectra of NaYF_4_:Yb/Er and NaYF_4_:Yb/Er doped with Gd^3+^ normalized by the respective peaks at approximately 650 nm.

Our samples were also characterized by XRD. The XRD pattern of NaYF_4_:Yb/Er in Figure 3a shows that all diffraction peaks can be attributed to the hexagonal structure of NaYF_4_ (JCPDS No. 16-0334). Upon doping with Gd^3+^, the peaks shift toward lower diffraction angles. This shift results from lattice expansion due to the substitution of Y^3+^ by Gd^3+^. Additionally, the diffraction peaks become broader after Gd^3+^ doping, as shown in Figure 3b, implying a reduction in average crystallite size, which agrees with the STEM results. Using the XRD data and the Bragg equation 2d sinθ = nλ, we determined the lattice parameters for the hexagonal β-NaYF_4_ phase. Lattice parameters: a = b = 5.96 Å, c = 3.53 Å (Gd-free), yielding a unit cell volume of ~108.6 Å^3^. For the 6% Gd-doped sample, the lattice constants are estimated to be a = b = 5.995 Å and c = 3.553 Å, yielding a unit cell volume of ~110.5 Å^3^. The crystallite size, calculated using the Scherrer equation D = Kλ/(β cosθ), decreases from ~55 nm (Gd-free) to ~30nm (6% Gd). Although widely used for synthesizing fluoride-based upconversion nanoparticles (UCNPs), the thermal decomposition method required that the temperature and time at each stage be carefully optimized. These parameters were determined as the key process parameters in this study. The initial low-temperature stage facilitates the removal of moisture and the formation of metal-oleate complexes. A specific high-temperature stage (290–310 °C) maintained for 1–1.5 h is critical for forming the pure hexagonal phase and controlling the nanocrystal size and morphology (ellipsoidal). Omitting or altering any of these stages can result in phase impurities, irregular morphology, or a significant deterioration in luminescence performance.

Furthermore, the changes in the upconversion luminescence spectra before and after Gd^3+^ doping were investigated. The upconversion emission spectra of NaYF_4_:Yb/Er and Gd^3+^-doped NaYF_4_:Yb/Er are presented in Figure 3c, from which Gd^3+^ doping was found to enhance the upconversion luminescence. The green-to-red emission ratio of NaYF_4_:Yb/Er/Gd is higher than that of NaYF_4_:Yb/Er, as shown in Figure 3d. With the specific doping method and proportion, this material exhibits a significantly higher ratio of green-to-red emission intensity compared to its Gd^3+^-free counterpart. This implies an effective purification of the output color, resulting in a more vibrant and intense green signal. Consequently, the legibility and anti-counterfeiting efficacy of the security labels are greatly enhanced. It is worth noting that, in the optical response process, the emitted light intensity largely depends on electron transitions, lattice vibrations, and energy transfer among different degrees of freedom. Crystal lattice distortion can alter the arrangement and spatial distribution of electrons, thereby affecting the magnetic, optical, and conductive properties of the material. Introducing local lattice distortion through doping can achieve multiple effects: it alters the crystal field symmetry around rare-earth ions, partially lifts the parity restriction on f-f transitions, and greatly enhances the probability of these transitions [43]. In addition, the Gd-free particles have an average size of approximately 60 nm, whereas the Gd-containing particles have an average size of approximately 30 nm. Because particle size strongly affects surface-related nonradiative relaxation, scattering, absorption, energy migration, and upconversion efficiency, the observed differences in luminescence cannot be uniquely attributed to Gd^3+^ doping.

### 3.2. The Influence of Gd^3+^ Doping Concentration on the Luminescence Intensity of NaYF_4_:Yb/Er/Gd

**Figure 4:**
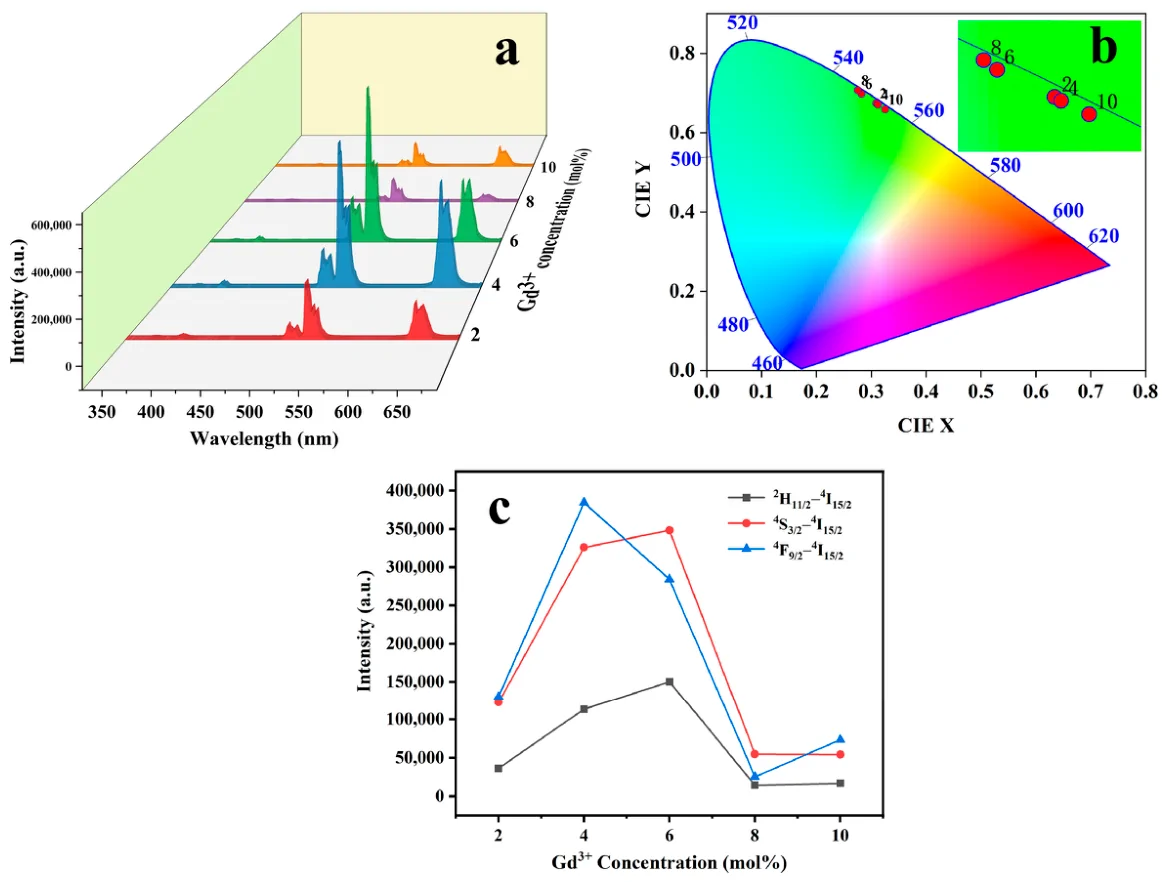
(**a**) Upconversion emission spectra of the NaYF_4_:Yb/Er/Gd nanoparticles doped with different Gd^3+^ concentrations (2, 4, 6, 8, and 10 mol%). (**b**) CIE diagram of NaYF_4_:Yb/Er/Gd nanoparticles. (**c**) The Gd-concentration dependence of upconversion intensity.

The effect of Gd^3+^ dopant concentration on enhancing the upconversion luminescence intensity was investigated. The emission spectra for nanoparticles doped with different Gd^3+^ concentrations (2, 4, 6, 8, and 10 mol%) are shown in Figure 4a. The upconversion luminescence intensity increases with increasing Gd^3+^ doping concentration. Among the tested concentrations, the upconversion luminescence emission intensity is highest at 6 mol% Gd^3+^. When the doping concentration increases to 8 and 10 mol%, the upconversion luminescence intensity decreases. The CIE chromaticity coordinates were derived from the upconversion luminescence spectra to evaluate the color purity of the emission. The color coordinate changes with different Gd^3+^ concentrations are presented in the CIE diagram of Figure 4b, where Gd^3+^ doping concentrations of 2, 4, 6, 8, and 10 mol% are indicated by labels 2–10, respectively. The integrated UC emission intensities of the green and red bands as a function of Gd^3+^ concentration are shown in Figure 4c. Both green and red emissions first increase with Gd^3+^ doping and then decrease at higher concentrations. The doping concentration of Gd^3+^ ions have a significant impact on the luminescence performance of the UCNPs. This study demonstrates that optimal Gd^3+^ doping enhances the emission intensity of NaYF_4_:Yb/Er/Gd nanoparticles.

### 3.3. Upconversion Mechanism of Gd^3+^-Doped NaYF_4_:Yb/Er

**Figure 5:**
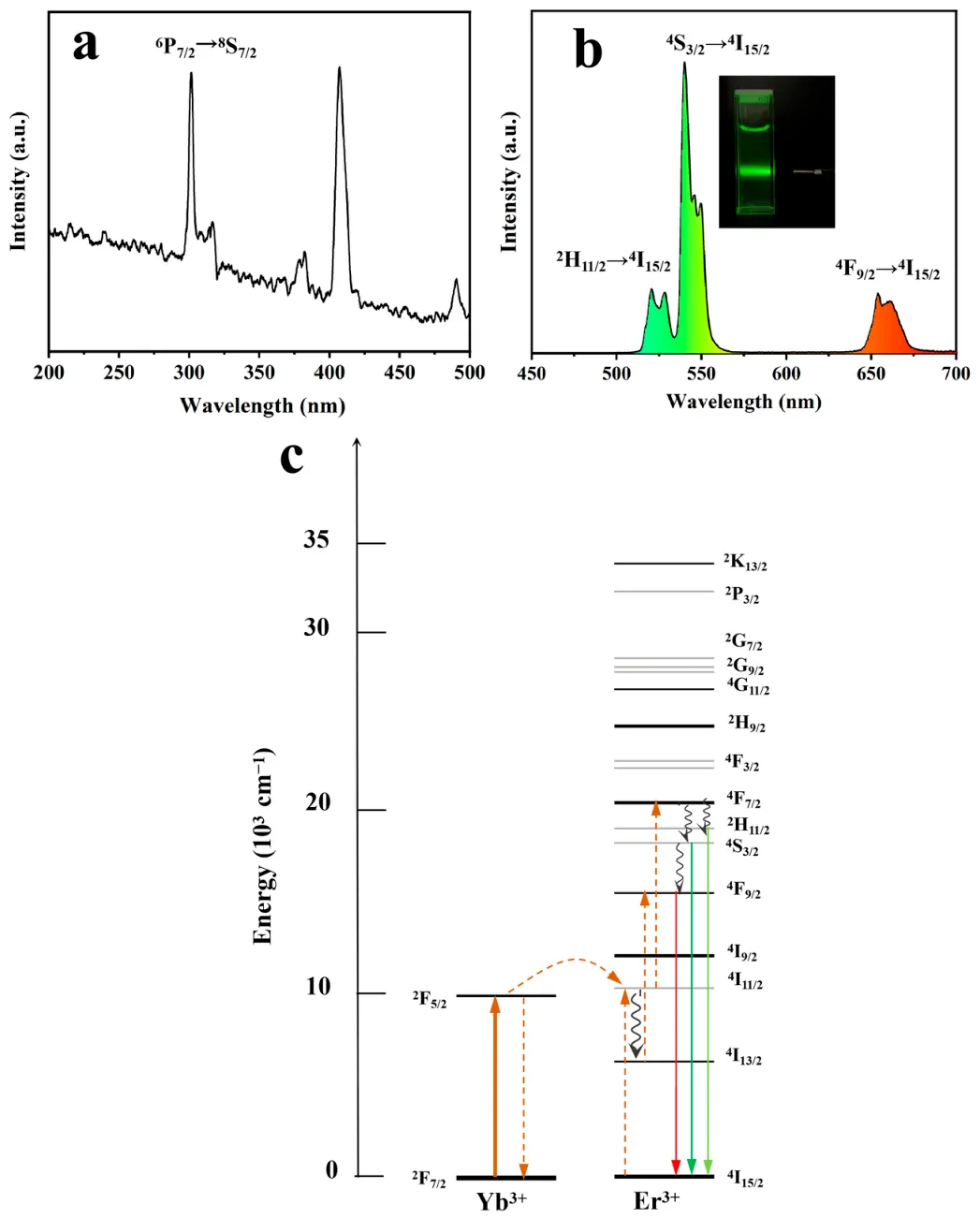
(**a**) Luminescence spectra of NaYF_4_:Yb/Er/Gd nanoparticles dispersed in cyclohexane (the range of 200–500 nm). (**b**) Luminescence spectra of NaYF_4_:Yb/Er/Gd nanoparticles dispersed in cyclohexane (the range of 500–700 nm). The inset shows the corresponding luminescence photographs of NaYF_4_:Yb/Er/Gd in solution under 980 nm laser excitation. (**c**) Energy transfer mechanisms under 980 nm diode laser excitation of NaYF_4_:Yb/Er nanoparticles. Orange solid arrows represent photon excitation. Orange dashed arrows represent energy transfer. The red and green solid arrows indicate the radiative emissions at different wavelengths.

While numerous reports have focused on the influence of Yb^3+^ on upconversion materials, the impact of Gd^3+^ doping on upconversion has been comparatively underexplored. Therefore, a detailed analysis was performed of the luminescence spectra and the associated mechanisms. Figure 5a,b show the upconversion luminescence spectrum of NaYF_4_:Yb/Er/Gd nanoparticles in cyclohexane under 980 nm laser excitation with a power density of 7.1 W/cm^2^. The UC emission spectra of NaYF_4_:Yb/Er/Gd nanoparticles under 980 nm excitation in the range of 200–500 nm are displayed in Figure 5a. The characteristic emissions of ^6^P_7/2_→^8^S_7/2_ (312 nm, Gd^3+^) are presented clearly [44]. However, they belong to ultraviolet light, which does not affect the UC in the visible-light region. The UC emission spectra of NaYF_4_:Yb/Er/Gd nanoparticles under 980 nm excitation in the range of 500–700 nm are displayed in Figure 5b. Three emission peaks appear at 524, 545, and 660 nm. The green peak at 524 nm corresponds to the ^2^H_11/2_→^4^I_15/2_ transition of Er3+, and the peak at 545 nm corresponds to the ^4^S_3/2_→^4^I_15/2_ transition. The red emission at 660 nm arises from the ^4^F_9/2_→^4^I_15/2_ transition. The strongest intensity is observed at 545 nm. Additionally, the solution containing NaYF_4_:Yb/Er/Gd nanoparticles emits visible green light under 980 nm excitation, as shown in the inset of Figure 5b. This confirms that the synthesized nanoparticles can emit strong green upconversion luminescence upon lanthanide ion doping. Figure 5c shows the upconversion mechanism for NaYF_4_:Yb/Er under excitation by a 980 nm laser. The photon excitation and energy transfer are represented by the orange solid and dashed arrows, respectively. The radiative emissions at different wavelengths are indicated by the red and green solid arrows. For the co-doping of Yb^3+^ and Er^3+^, the energy level of the Yb^3+^ excited state ^2^F_5/2_ is very close to that of the neighboring Er^3+^ excited state ^4^I_11/2_, allowing for energy transfer between them. Generally, the photon-excited electron transition occurs between the ground state ^2^F_7/2_ and the excited state ^2^F_5/2_ of Yb^3+^, and the electrons can then be transferred to the neighboring state ^4^I_11/2_ of Er^3+^. The electrons in state ^4^I_11/2_ transition to the high state ^4^F_7/2_ through energy transfer. Electrons in the high state ^4^F_7/2_ cannot directly transition to the lower state. These transitions require mediating states ^2^H_11/2_ and ^4^S_3/2_, which are realized by multiple phonon relaxation. Therefore, electronic transitions can occur from the states ^2^H_11/2_ and ^4^S_3/2_ to the lowest state ^4^I_15/2_, emitting green light. Similarly, the electrons in state ^4^I_11/2_ can also transit to the lower state ^4^I_13/2_ by multiple phonon relaxations, followed by energy transfer to the higher state ^4^F_9/2_. In this respect, red light is emitted during the transition from state ^4^F_9/2_ to state ^4^I_15/2_. Additionally, the electrons in state ^4^I_11/2_ come not only from state ^2^F_5/2_, as mentioned above, but can also be realized from the transition between states ^4^I_15/2_ and ^4^I_11/2_ of Er^3+^. However, the contribution of the latter is minimal.

**Figure 6:**
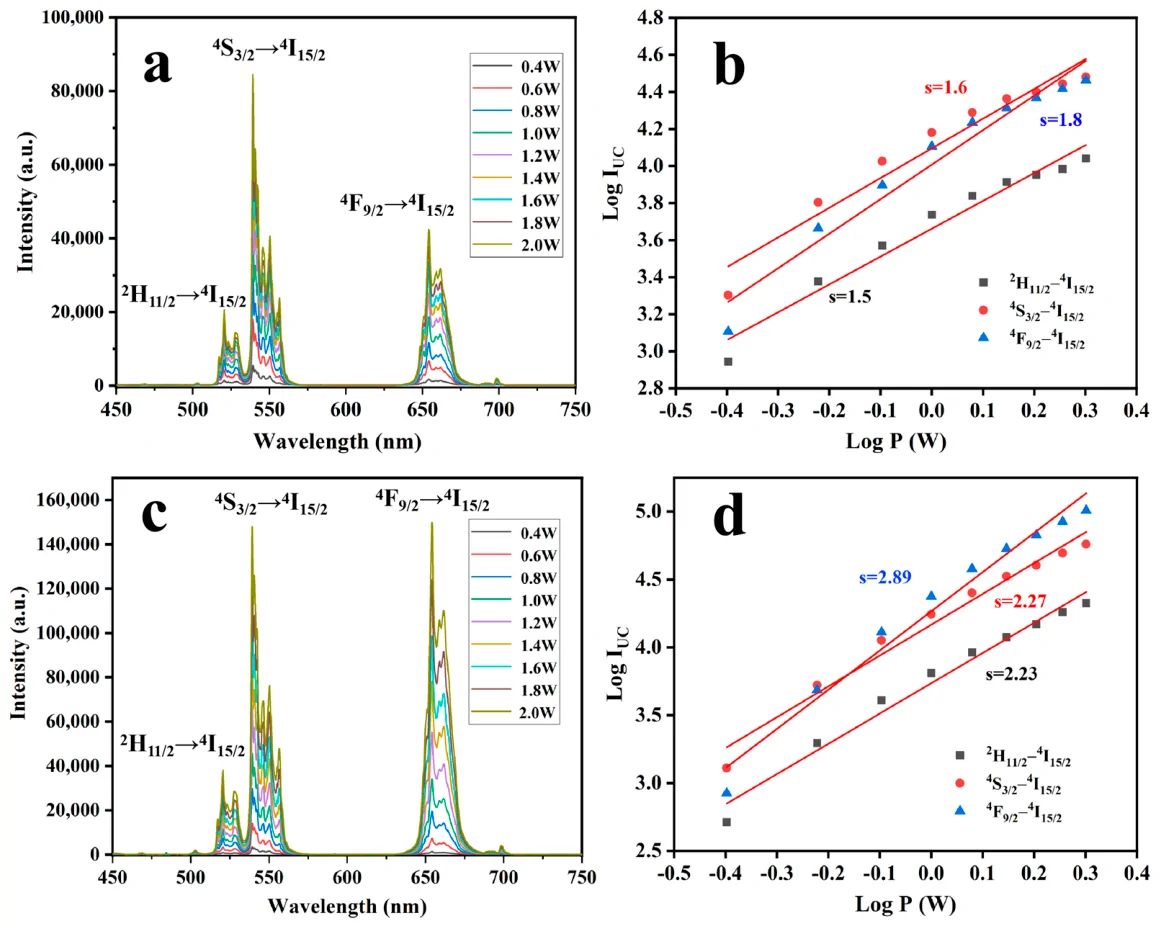
(**a**) Upconversion luminescence of NaYF_4_:Yb/Er nanoparticles with different exciting powers. (**b**) Pump power dependence of the green and red emission of NaYF_4_:Yb/Er nanoparticles. (**c**) Upconversion luminescence of NaYF_4_:Yb/Er/Gd nanoparticles with different exciting power. (**d**) Pump power dependence of the green and red emission of NaYF_4_:Yb/Er/Gd nanoparticles.

We compared the number of photons before and after Gd doping to further investigate the mechanism behind the enhanced luminescence intensity. Usually, in Yb-Er systems, the upconversion mechanism is based on either two- or three-photon absorption. In order to obtain the excitation laser power-dependent luminescence spectra, the emission spectra of NaYF_4_:Yb/Er nanoparticles and NaYF_4_:Yb/Er/Gd nanoparticles are recorded using the fluorescence spectrometer. Earlier studies on upconversion particles demonstrated a biphotonic power dependence, with the upconversion emission intensity following IUC∝Pn, where n is the number of pump photons needed to excite Er^3+^ ions from the ground state to the emitting state. Accordingly, a plot of log IUC against log P provides a straight line with slope s. We employed a laser power between 0.4 W and 2.0 W. As shown in Figure 6a (NaYF_4_:Yb/Er) and Figure 6c (NaYF_4_:Yb/Er/Gd), the luminescence intensity of all the doped samples increases monotonically with the excitation power. The log–log plots of all the samples investigated here are given in Figure 6b (NaYF_4_:Yb/Er) and Figure 6d (NaYF_4_:Yb/Er/Gd). In our case, the slopes of two full green region transitions (524 nm and 545 nm corresponding to ^2^H_11/2_→^4^I_15/2_ and ^4^S_3/2_→^4^I_15/2_) and a red region transition (660 nm corresponds to ^4^F_9/2_→^4^I_15/2_) are estimated for NaYF_4_:Yb/Er (78:20:2) and NaYF_4_:Yb/Er/Gd (72:20:2:6). It is pertinent to note here that, for NaYF_4_:Yb/Er (78:20:2), the slope value is less than 2, indicating that the upconversion process is a two-photon phenomena. As shown in Figure 6d, the observed changes suggest that Gd^3+^ doping may introduce local lattice distortion, which in turn enhances multiphoton excitation. This explains why the doping of Gd^3+^ can enhance the emission intensity.

### 3.4. Anti-Counterfeiting Application of Gd^3+^-Doped NaYF_4_:Yb/Er

**Figure 7:**
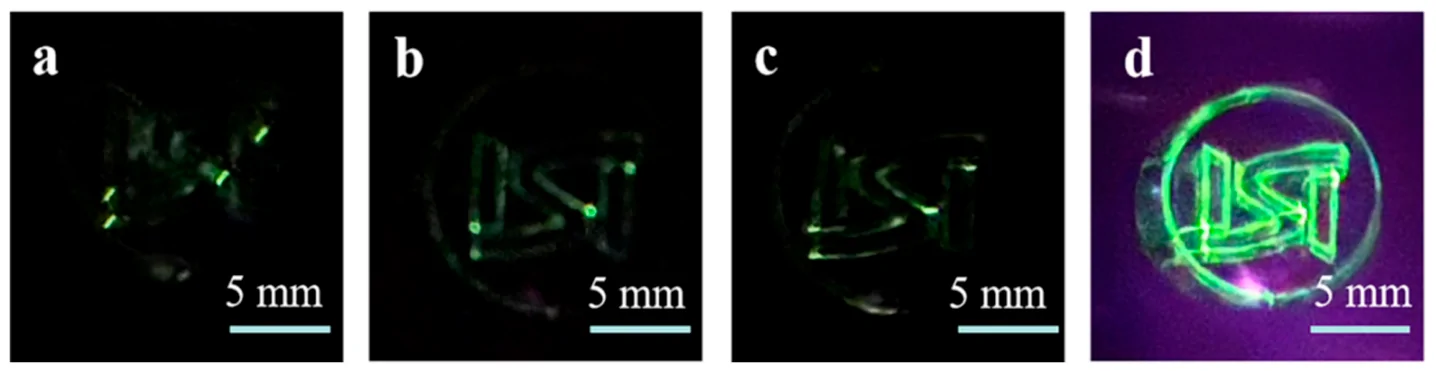
Changes in luminescence with the change in UCNP concentration: (**a**) 0.2, (**b**) 0.5, (**c**) 0.8, and (**d**) 1.6 mg/mL.

NaYF_4_:Yb/Er/Gd nanoparticles are typically used in anti-counterfeiting applications. To obtain a uniform and high-luminescence pattern, the ink must be composed of fluorescent nanoparticles that possess good solubility and high luminescence intensity. The pattern was designed on a computer and printed using an inkjet printer. The nanoparticles should be 50 times smaller than the printer nozzle diameter to prevent it from being closed and blocked. The sample size was 1 cm. As shown in Figure 7, photochromism can be observed in the area of the prepared NaYF_4_:Yb/Er/Gd nanoparticles after 980 nm light irradiation for 3 s. Therefore, a green color can be observed in the pattern, corresponding to the upconversion emission spectrum of the 980 nm irradiation. To verify the luminescence uniformity of printed patterns, the variance of the luminescence intensity in a ring printing area with different concentrations varying from 0.2 to 1.6 mg/mL was tested under 980 nm excitation with a power density of ~80 mW/cm^2^. The prepared nanoparticles were found to be suitable for printing complex patterns. Moreover, this method is universally applicable to various functional materials.

## 4. Conclusions

In this work, the NaYF_4_:Yb/Er nanoparticles were transformed from irregular shapes to a highly uniform shape by doping with an ultralow concentration of Gd^3+^ ions, yielding particles of ~30 nm that demonstrated excellent dispersity and were used for the preparation of anti-counterfeiting inks. The uniform morphology of the nanoparticles is crucial for formulating stable and efficient inks. Combined with an appropriate ink ratio, it effectively avoids clogging the nozzle and enables high-resolution printing. The high upconversion luminescence of the NaYF_4_:Yb/Er/Gd nanoparticles was demonstrated. Furthermore, a systematic study on the mechanism was conducted. Doping with Gd^3+^ to replace Y^3+^ ions introduces local lattice distortion, which directly enhances the luminescence intensity. Our work provides excitation-power-dependent log–log analysis demonstrating that Gd^3+^ incorporation modifies the effective photon number of the green and red upconversion channels. This provides direct experimental evidence that Gd^3+^ doping induces local lattice distortion, thereby enhancing multiphoton excitation. Our materials were engineered to possess a specific composition, uniform ellipsoidal morphology, and high green-to-red emission ratio. These features meet the requirements for high-resolution inkjet anti-counterfeiting applications. Prior anti-counterfeiting demonstrations using UCNPs have generally employed larger particles or different deposition methods. Our work demonstrates that Gd^3+^-mediated size reduction to ~30 nm enables stable inkjet-printed patterns with improved dispersion stability, which is a practical engineering contribution distinct from previous reports. This work not only provides an enhanced light emission upconversion nanomaterial but also reveals its optical mechanism for Gd doping, providing theoretical and experimental insights for rational design and expanded applications of novel luminescent materials.

## Figures and Tables

**Table 1:** Elemental composition of the as-prepared UCNP.

Element	Concentration (mg/L)	Atomic Weight (g/mol)	Mole Number (×10^−6^ mol)	Mole Percentage
Y	~51.5	88.906	579	~71.35%
Yb	~28.0	173.045	162	~19.93%
Er	~2.8	167.26	16.7	~2.06%
Gd	~8.5	157.25	54.1	~6.66%

## Data Availability

The raw data supporting the conclusions of this article will be made available by the authors upon request.
